# Cisplatin-Based Chemotherapy versus Cetuximab in Concurrent Chemoradiotherapy for Locally Advanced Head and Neck Cancer Treatment

**DOI:** 10.1155/2014/904341

**Published:** 2014-07-06

**Authors:** Ming-Hung Hu, Ling-Wei Wang, Hsueh-Ju Lu, Pen-Yuan Chu, Shyh-Kuan Tai, Tsung-Lun Lee, Ming-Huang Chen, Muh-Hwa Yang, Peter Mu-Hsin Chang

**Affiliations:** ^1^Division of Hematology and Oncology, Department of Medicine, Taipei Veterans General Hospital, No. 201, Section 2, Shih-Pai Road, Taipei 112, Taiwan; ^2^Faculty of Medicine, National Yang Ming University, No. 155, Section 2, Linong Street, Taipei 112, Taiwan; ^3^Division of Hematology and Oncology, Department of Medicine, Cardinal Tien Hospital, No. 362, Zhongzheng Road, New Taipei City 231, Taiwan; ^4^Cancer Center, Taipei Veterans General Hospital, No. 201, Section 2, Shih-Pai Road, Taipei 112, Taiwan; ^5^Division of Hematology and Oncology, Department of Medicine, Show Chwan Memorial Hospital, No. 542, Section 1, Zhongshan Road, Changhua City, Changhua County 500, Taiwan; ^6^Department of Otolaryngology, Taipei Veterans General Hospital, No. 201, Section 2, Shih-Pai Road, Taipei 112, Taiwan; ^7^Institute of Clinical Medicine, National Yang Ming University, No. 155, Section 2, Linong Street, Taipei 112, Taiwan

## Abstract

*Background and Purpose*. This study aimed to analyze survival, clinical responses, compliance, and adverse effects in locally advanced head and neck cancer (LAHNC) patients treated with split-dose cisplatin-based concurrent chemoradiation therapy (SD-CCRT) or cetuximab with concurrent radiation therapy (BioRT). *Materials and Methods*. We retrospectively evaluated 170 LAHNC patients diagnosed between January 1, 2009, and July 31, 2012: 116 received CCRT and 54 received BioRT. *Results*. Complete response rates were similar in the SD-CCRT and BioRT groups (63.8% versus 59.3%; *P* = 0.807), and locoregional relapse rates were 18.1% and 13.0%, respectively (*P* = 0.400). The 3-year relapse-free survival rate was 65.8% in the SD-CCRT group and 65.5% in the BioRT group, respectively (*P* = 0.647). The 3-year overall survival rate was 78.5% in the SD-CCRT group and 70.9% in the BioRT group, respectively (*P* = 0.879). Hematologic side effects were significantly more frequent in the SD-CCRT than in the BioRT group. Mucositis frequency was similar. *Conclusions*. Primary SD-CCRT and BioRT both showed good clinical response and survival. Hematologic toxicities were more frequent, but tolerable, in the SD-CCRT group. Both groups showed good compliance.

## 1. Introduction

Treatment is complex for patients with head and neck cancer, which requires a multidisciplinary team with expertise in the management of this condition. In locally advanced head and neck cancer (LAHNC), primary concurrent chemoradiation therapy (CCRT) remains the current standard organ-preservation treatment for resectable disease [1, 2]. For unresectable disease, CCRT results in an optimal locoregional control and has become a treatment cornerstone [1, 2]. With regard to combination chemotherapy, 100 mg/m^2^ cisplatin, triweekly, has been regarded as standard during the past decade [1, 2]. However, a gradual increase in poor compliance rates was noted. Several studies have aimed at identifying a chemotherapy regimen with similar efficacy but better tolerance [3–6]. Combination with targeted agents such as antiepidermal growth factor receptor (EGFR) is one alternative. Cetuximab, a monoclonal antibody against EGFR, could enhance the cytotoxic effects of radiation and is associated with a much lower rate of acute complications, including acute stomatitis and cytopenia. Bonner et al. demonstrated that cetuximab with concurrent radiotherapy (BioRT) significantly improved locoregional control, progression-free survival, and overall survival compared to radiotherapy alone [7, 8]. Furthermore, BioRT did not have an adverse impact on the timely completion of definitive radiotherapy [7]. Although its efficacy compared to CCRT is still controversial [9], BioRT has gradually become an alternative for patients with LAHNC who are ineligible for CCRT because of advanced age or comorbidities.

Split-dose cisplatin in CCRT (SD-CCRT) is thought to be better tolerated than triweekly cisplatin and increase compliance [5, 6]. Further, 5-fluorouracil (5-FU) and hydroxyurea have both been established as radiosensitizers [10, 11]. Hydroxyurea is thought to modulate the activity of 5-FU by depleting cellular deoxyuridine monophosphate (dUMP), thus potentiating the binding of the 5-FU metabolite 5-FdUMP to its target enzyme thymidylate synthase [12, 13]. Interestingly, these studies of the cisplatin, 5-FU, and hydroxyurea regimen (CFHx) showed a relatively good toxicity profile and good compliance when using SD-CCRT [5, 6].

The aim of this study was to compare treatment outcomes between SD-CCRT and BioRT. Several factors, including response, disease-free survival, overall survival, treatment compliance, and adverse effects, were analyzed.

## 2. Materials and Methods

### 2.1. Patient Selection

This retrospective study enrolled adult patients (≥18 years) with LAHNC who were diagnosed and treated in Taipei Veterans General Hospital between January 1, 2009, and July 31, 2012. All patients were diagnosed with nonmetastatic, untreated squamous cell carcinoma of the oral cavity, oropharynx, hypopharynx, or larynx. Disease staging was performed in accordance with the American Joint Committee on Cancer 2009 criteria. The pretreatment workup included history taking and physical examination, endoscopic evaluation, dental evaluation, plain radiography of the chest, head and neck computed tomography (CT) or magnetic resonance imaging (MRI), and bone scanning. When distant metastasis was suspected and could not be completely ruled out in selected locally advanced patients, PET-CT was performed before treatment. Only those without distant metastasis received SD-CCRT or BioRT. All patients were evaluated by a multidisciplinary team, including a medical oncologist, radiation oncologist, and surgeons, before treatment initiation. A multidisciplinary conference discussion was held to determine whether patients had unresectable or inoperable disease, after which patients were assigned to curative nonsurgical management with either SD-CCRT or BioRT. The study has been approved by the local ethical committee.

### 2.2. Combination Treatment

The CFHx regimen consisted of 2 courses of 20 mg/m^2^ cisplatin on days 1–4, 600 mg/m^2^ 5-FU on days 1–4, and 500 mg hydroxyurea twice daily for 11 doses, every 3 weeks. For patients with renal insufficiency, hearing impairment, or advanced age, cetuximab administration was initiated 1 week before radiation at a loading dose of 400 mg/m^2^ over a period of 120 minutes, followed by weekly 60-minute infusions of 250 mg/m^2^ for the duration of radiotherapy.

Before radiotherapy, all patients were immobilized using a thermoplastic mask and shoulder fixation device. CT simulation was performed at a 3 mm slice thickness. Contrast material was intravenously injected for all patients without contraindications. In general, radiotherapy was performed using the intensity-modulated radiotherapy technique. Treatment planning was performed using the Eclipse system, version 6 (Varian Medical Systems, Inc., Palo Alto, CA, USA). The gross tumor volume (GTV) was defined as any visible tumor on imaging studies and/or physical examination. The high-risk clinical tumor volume (CTV_H) encompassed the GTV with a 5–10 mm margin around it, including the nodal regions in the neck at Levels I–IV. The low-risk CTV (CTV_L) included the clinically uninvolved contralateral neck and base of the skull. The retropharyngeal region was also included as part of the CTV in patients who presented with clinically involved neck nodes as well as in those who had primary oropharyngeal or hypopharyngeal tumors. An intermediate risk CTV (CTV_M) was generated by the treating physician for areas with a risk that was intermediate between that considered for CTV_L and CTV_H. The planning target volumes (PTV_H, M, and L) encompassed the corresponding CTVs plus a 3 mm margin. The PTV was modified if indicated (e.g., in cases where it was close to critical organs).

The simultaneous integrated boost technique was used for two-step planning. In the first step, 56 Gy/28 fractions were administered to the PTV_H and 50.4 Gy/28 fractions were administered to the PTV_M and PTV_L. In the second step, 14 Gy/7 fractions were delivered to the PTV_H and 12.6 Gy/7 fractions were delivered to the PTV_M. The total prescription dose to the PTV_H was 70 Gy/35 fractions. Prescription doses to the PTV_M and PTV_L were 63 Gy/35 fractions and 50.4 Gy/28 fractions, respectively. At least 95% of the PTVs was covered by the prescription doses. Radiation (6-MV photons) via 7 intensity-modulated fields was delivered at the rates of 1 fraction per day and 5 fractions per week, using a Varian 2100CD linear accelerator (Varian Medical Systems, Inc.).

### 2.3. Surveillance

After treatment completion, patients were typically followed up every month in the first year and every 3–6 months thereafter. CT or MRI was performed at least 2 months after completion of radiotherapy. Response was assessed according to Response Evaluation Criteria in Solid Tumors v1.1 [14]. Data on adverse events were retrieved from the medical charts at admission and during outpatient clinic follow-up, and adverse events were recorded according to the Radiation Therapy Oncology Group (RTOG) criteria for radiation effects and the RTOG Cooperative Group Common Toxicity Criteria for systemic effects [15].

### 2.4. Statistical Analysis

Categorical variables were compared between patients who received SD-CCRT or BioRT using the *χ* ^2^ test or the Fisher exact test, and the log-rank test was used to compare survival curves. A *P* value of <0.05 was regarded as statistically significant in 2-sided tests. Kaplan-Meier methods were used to evaluate time to disease recurrence or death. Cox regression was used for univariate and multivariate analyses to determine the potential risk factors associated with disease-free survival and overall survival. All statistical analyses were performed using SPSS statistical software version 18 (SPSS, Chicago, IL, USA).

## 3. Results

### 3.1. Patients Characteristics

Between January 1, 2009, and July 31, 2012, 170 patients were enrolled in this study. 116 patients received CFHx-based SD-CCRT and 54 received BioRT. Patient characteristics are listed in Table 1. The median ages of patients in the SD-CCRT and BioRT groups were 55 (33–74) and 78 (46–94) years, respectively (*P* = 0.000). Patients in the SD-CCRT group had a more advanced stage of disease (*P* = 0.002). The median follow-up time was 22.5 months. The primary tumor site was significantly different between the two groups: oral cavity cancer accounted for 32.8% of cases in the SD-CCRT group and only 9.3% of cases in the BioRT group (*P* = 0.000). In contrast, larynx cancer was observed in only 3.4% of cases in the SD-CCRT group but in 29.6% of cases in the BioRT group (*P* = 0.000) (Table 1).

### 3.2. Compliance with Treatment

The basic treatment characteristics are listed in Table 2. In total, 84.5% of patients in the SD-CCRT group completed 2 cycles of chemotherapy. In the SD-CCRT group, 90.8% of patients received at least 6 cycles of cetuximab infusion (Table 3). The median RT doses per protocol for the PTV_H were 70 Gy (range 64–74) and 70 Gy (range 64–72) in the SD-CCRT and BioRT groups, respectively (*P* = 0.268). The median RT intervals per protocol were 46.0 days (39–78) and 46.0 days (35–62) in the SD-CCRT and BioRT groups, respectively (*P* = 0.061). Only 17 patients (14.6%) in the SD-CCRT group and 7 patients (12.9%) in the BioRT group had interruptions during radiotherapy. The major cause of radiation interruption was neutropenia (8 patients, 47.1%) in the SD-CCRT group and allergic reaction to cetuximab (4 patients, 57.1%) in the BioRT group. Among these 17 patients, 4 patients in the SD-CCRT group and 3 patients in the BioRT group did not complete radiotherapy as scheduled, due to intolerable toxicity.

### 3.3. Efficacy

**Figure 1:**
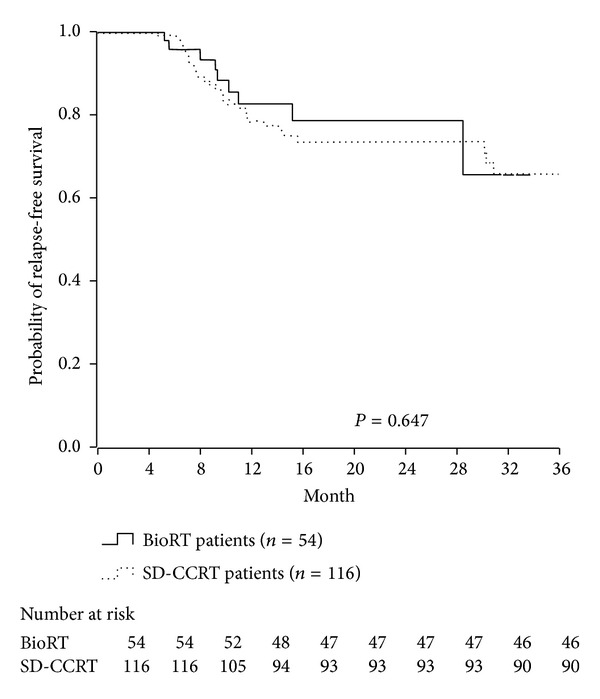
3-year relapse-free survival between BioRT and SD-CCRT group. 3-year RFS is 65.8% in SD-CCRT group versus 65.5% in BioRT group (*P* = 0.647).

**Figure 2:**
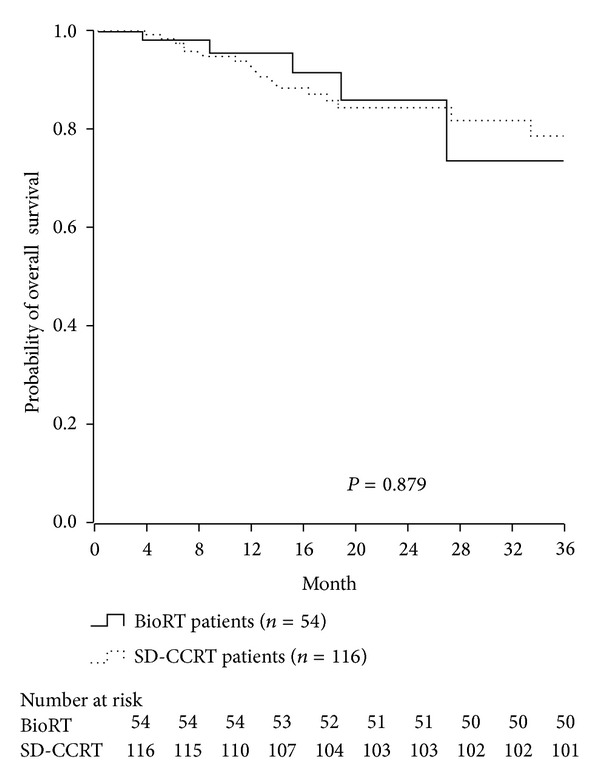
3-year overall survival between BioRT and SD-CCRT group. 3-year OS is 78.5% in SD-CCRT group versus 70.9% in BioRT group (*P* = 0.879).

The complete response rate was similar in the SD-CCRT and BioRT groups (63.8% versus 59.3%; *P* = 0.807). After a median follow-up of 22.5 months, locoregional relapse was noted in 18.1% of patients in the SD-CCRT group and in 13.0% of patients in the BioRT group (*P* = 0.400), whereas distant metastasis was noted in 6.9% of patients in the SD-CCRT group and 3.7% of patients in the BioRT group (*P* = 0.410) (Table 4). The 3-year relapse-free survival rate was 65.8% in the SD-CCRT group and 65.5% in the BioRT group (*P* = 0.647; Figure 1). The 3-year overall survival rate was 78.5% in the SD-CCRT group and 70.9% in the BioRT group (*P* = 0.879; Figure 2).

### 3.4. Factors Associated with Survival

We analyzed age, radiotherapy dose, induction chemotherapy, cancer stage, and primary tumor location as prognostic factors for survival in all patients. Univariate analysis revealed that patients <60 years old (hazard ratio [HR]: 2.186, 95% confidence interval [CI]: 1.049–4.556; *P* = 0.037) and those with a more advanced tumor stage (HR: 2.238, 95% CI: 1.106–3.723; *P* = 0.010) had a significantly poor 3-year relapse-free survival rate. However, multivariate analysis revealed that none of these factors were associated with relapse-free survival. In our study, 42 patients with SD-CCRT and 26 patients with BioRT had positive smoking history. We also analyzed smoking as a prognostic factor regarding patient's survival; however, smoking is not significantly related to survival. Neither were any of the other factors significantly associated with overall survival.

### 3.5. Safety

Hematologic side effects were significantly more frequent in the SD-CCRT group than the BioRT group (neutropenia 45.7% versus 18.5%, anemia 80.1% versus 48.1%, and thrombocytopenia 98.3% versus 26.0%). In addition, radiation dermatitis occurred more frequently in the SD-CCRT group (69.8%) than in the BioRT group (48.1%; *P* = 0.033). The incidence of mucositis was similar in the two groups (88.8% versus 87.0%; *P* = 0.747). Skin acne related to cetuximab treatment was noted in 68.5% of patients in the BioRT group. Other nonhematologic toxicities including nausea, vomiting, diarrhea, and acute kidney injury were similar in both groups (Table 4).

## 4. Discussion

In this study, we demonstrated that both SD-CCRT and BioRT were tolerable and effective. There were differences in patient characteristics between the two groups. These differences can be partially explained by the fact that in Taiwan cetuximab is covered by National Health Insurance for elderly patients (age > 70) in cases of oropharyngeal, hypopharyngeal, and laryngeal squamous cell carcinoma when used with concurrent radiotherapy. Therefore, the proportion of elderly patients was higher in the BioRT group, while fewer patients with oral cavity cancer received BioRT. Another significant difference in characteristics was in the disease stage. This implies that physicians preferred CCRT to BioRT for patients with advanced disease.

Cisplatin-based CCRT is the standard treatment strategy for unresectable or inoperable LAHNC. Although both excellent response and organ preservation have been noted, poor compliance was an important issue in the past decades. In large randomized studies, grade 3 and 4 hematotoxicity was observed in 47% of the patients receiving CCRT. Around 30–40% of other acute nonhematologic toxicities, such as stomatitis and pharyngeal/esophageal dysfunction, have also been observed [1]. In the traditional CCRT regimen, only 50–70% of patients could complete all three planned doses of cisplatin, resulting in a lower compliance rate if previous induction chemotherapy was performed [2, 9]. In our study, grade 3 and 4 neutropenia was observed in 17% of patients in the SD-CCRT group, which was much lower than that with traditional cisplatin-based CCRT. No grade 5 neutropenia was observed. Importantly, almost all patients were able to receive two complete courses of CFHx, even though 56% of patients had received induction chemotherapy before CCRT. In the BioRT group, most patients completed the entire course of cetuximab (≥6 cycles) treatment during radiation, as in published reports. Although only two cycles of CFHx during radiation were included in our CCRT plan, the median relapse-free and overall survival were similar to those in previous studies [3, 9], likely because most patients received induction chemotherapy in the SD-CCRT group and 64.7% of patients could receive the cumulative dosage of cisplatin of 200 mg/m^2^. In addition, even after induction chemotherapy, most patients (84.5%) could complete 2 full courses of chemotherapy during the planned radiotherapy schedule. Induction chemotherapy was given in 65 patients (56.0%) in SD-CCRT group and 8 patients (14.8%) in BioRT group. The proportion of induction chemotherapy was much higher in SD-CCRT group. It suggests that induction chemotherapy is more commonly used in patients with more advanced disease. However, our multivariate analysis reveals that induction chemotherapy is not prognostic to patient's survival. Recent studies also showed that induction chemotherapy cannot improve the survival [9, 16]. The possible explanation may be due to the diversity of tumor locations among enrolled patients and selection bias in regard to induction chemotherapy for more advanced disease.

In the survival analysis, it is not surprising that disease stage had an impact on relapse-free survival. Younger patients (<70 years) had inferior relapse-free survival in our study probably because of their more advanced disease stage. This could also explain why both factors were insignificant in multivariate analysis. There were no significant prognostic factors for overall survival, neither disease status nor age. This may be because of the high noncancer-related mortalities (50%), given the advanced age of patients in the BioRT group. In the subgroup analysis for SD-CCRT group, stage is indeed the significant prognostic factor regarding survival (HR 4.608, 95% CI 1.16 to 10.31; *P* = 0.026). Such significance cannot be shown in the BioRT group (HR 0.864, 95% CI 0.24–3.115; *P* = 0.823). Other factors did not contribute to survival in both groups. According to previous literatures, three-year survival rate in locally advanced head and neck cancers reached 60~70% [16, 17]. With the improvement of concurrent chemoradiotherapy and management of adverse effect, the survival rate could be better than before.

Most patients completed radiotherapy as scheduled. Only 17 patients (14.6%) in the SD-CCRT group and 7 patients (12.9%) in the BioRT group had interruptions during radiotherapy. The major cause of radiation interruption was neutropenia (8 patients, 47.1%) in the SD-CCRT group and allergic reaction to cetuximab (4 patients, 57.1%) in the BioRT group. Radiotherapy is a standard treatment for head and neck squamous cell carcinoma, which may escape RT-induced cell damage through the mechanism of accelerated repopulation. Therefore, compliance with radiotherapy is critical in patients with this progressive disease. Previous studies have demonstrated the importance of the time-dose relationship of radiotherapy [18–22]. In our study, in most patients the severity of acute toxicity during SD-CCRT or BioRT was manageable, and hence, all patients received uninterrupted radiotherapy.

There are some limitations in this study. Because of the selection bias for this retrospective analysis, the SD-CCRT could have been biased to do more poorly due to (1) higher proportion of oral cavity cancers, (2) higher proportion of induction chemotherapy which could have mitigated treatment compliance to concurrent chemoradiotherapy, and (3) higher proportion of stage 4a and stage 4b patients. The relatively short duration of median follow-up (13.05 months) in the cetuximab group could be a potential weakness of the analysis. HPV expression is a favored prognostic marker for head and neck cancer. However, in our hospital, HPV expression is only examined in patients with oropharyngeal or oral cavity cancer and with larger excision samples. In our study, there were 12 (21.2%) patients with oropharyngeal cancer and 4 (3.4%) patients with oral cavity cancer had been tested with HPV expression. Among these patients, only one patient with oropharyngeal cancer had HPV-positive. Owing to the limited HPV expression data, it is quite difficult to analyze the clinical impact in our study.

In conclusion, SD-CCRT and BioRT both demonstrated favorable clinical response, survival, compliance, and safety. Disease status seems to be the most important prognostic factor in LAHNC. Age-related comorbidities may influence overall survival and should be especially considered in the elderly.

## Figures and Tables

**Table 1:** The clinical characteristics of patients who received SD-CCRT and BioRT.

Patient characteristic	SD-CCRT (*n* = 116)	BioRT (*n* = 54)	*P* value
Sex, number of patients (%)			0.933
Male	112 (96.6%)	52 (96.3%)	
Female	4 (3.4%)	2 (3.7%)	
Median age, y	55 (33~74)	78 (46~94)	**0.000**
Age			**0.000**
<70	111 (95.7%)	9 (16.67%)	
≧70	5 (4.3%)	45 (83.33%)	
Median follow-up time (m)	19.11 (2.67~36.00)	13.05 (0.93~36.00)	0.288
Smoking	42 (48.8%)	26 (54.2%)	**0.592**
Primary tumor site	38 (32.8%)	5 (9.3%)	**0.000**
Oral cavity	37 (31.9%)	15 (27.8%)	
Oropharynx	37 (31.9%)	18 (33.3%)	
Hypopharynx	4 (3.4%)	16 (29.6%)	
Larynx			
Stage			**0.002**
2	11 (9.5%)	12 (22.2%)	
3	20 (17.2%)	16 (29.6%)	
4a	72 (62.1%)	26 (48.1%)	
4b	13 (11.2%)	0 (0.0%)	
Induction chemotherapy			**0.000**
Yes	65 (56.0%)	8 (14.8%)	
No	51 (44.0%)	46 (85.2%)	

SD-CCRT: split-dose cisplatin-based concurrent chemoradiation therapy.

BioRT: cetuximab with concurrent radiation therapy.

**Table 2:** Treatment outcome of patients who received SD-CCRT and BioRT.

Characteristic	SD-CCRT (*n* = 116)	BioRT (*n* = 54)	*P* value
Response			0.807
CR	74 (63.8%)	32 (59.3%)	
PR	34 (29.3%)	15 (27.7%)	
SD	5 (4.3%)	5 (9.3%)	
PD	3 (2.6%)	2 (3.7%)	
Local regional recurrence			0.400
Yes	21 (18.1%)	7 (13.0%)	
No	95 (81.9%)	47 (87.0%)	
Distant metastasis			0.410
Yes	8 (6.9%)	2 (3.7%)	
No	108 (93.1%)	52 (96.3%)	

CR: complete response; PR: partial response; SD: stable disease; PD: progressive disease.

**Table 3:** SD-CCRT/BioRT characteristics of patients received.

Characteristic	SD-CCRT (*n* = 116)	BioRT (*n* = 54)	*P* value
Cisplatin cumulative dose (mg/m^2^)			
≧200	75 (64.7%)		
<200	41 (35.3%)		
CFHx cycles			
2	98 (84.5%)		
1	18 (15.5%)		
Cetuximab cycles			
≧6		49 (90.7%)	
5		4 (7.4%)	
4		1 (1.9%)	
Median RT dose (Gy)	70 (64~74)	70 (64~72)	0.268
Median RT interval (days)	46.0 (35~79)	46.5 (34~62)	0.078

**Table 4:** Complications of patients who received SD-CCRT and BioRT.

Characteristic	SD-CCRT (*n* = 116)	BioRT (*n* = 54)	*P* value
Neutropenia			**0.003**
Grade 1~2	33 (28.5%)	10 (18.5%)	
Grade 3~4	20 (17.2%)	0 (0.0%)	
Anemia			**0.000**
Grade 1~2	88 (75.8%)	26 (48.1%)	
Grade 3~4	5 (4.3%)	0 (0.0%)	
Thrombocytopenia			**0.000**
Grade 1~2	114 (98.3%)	14 (26.0%)	
Grade 3~4	0 (0.0%)	0 (0.0%)	
Mucositis			0.747
Grade 1~2	47 (40.5%)	22 (40.7%)	
Grade 3~4	56 (48.3%)	25 (46.3%)	
Radiation dermatitis			**0.033**
Grade 1~2	79 (68.1%)	24 (44.4%)	
Grade 3~4	2 (1.7%)	2 (3.7%)	
Skin acne			**0.000**
Grade 1~2	0 (0.0%)	33 (61.1%)	
Grade 3~4	0 (0.0%)	4 (7.4%)	
Nausea/vomiting			0.142
Grade 1~2	6 (5.2%)	0 (0.0%)	
Grade 3~4	2 (1.7%)	0 (0.0%)	
Diarrhea			0.494
Grade 1~2	0 (0.0%)	0 (0.0%)	
Grade 3~4	1 (0.9%)	0 (0.0%)	
Acute kidney injury			0.089
Grade 1~2	6 (5.2%)	0 (0.0%)	
Grade 3~4	0 (0.0%)	0 (0.0%)	

## References

[1] Forastiere AA, Zhang Q, Weber RS (2013). Long-term results of RTOG 91-11: a comparison of three nonsurgical treatment strategies to preserve the larynx in patients with locally advanced larynx cancer. *Journal of Clinical Oncology*.

[2] Forastiere AA, Goepfert H, Maor M (2003). Concurrent chemotherapy and radiotherapy for organ preservation in advanced laryngeal cancer. *The New England Journal of Medicine*.

[3] Haraf DJ, Rosen FR, Stenson K (2003). Induction chemotherapy followed by concomitant TFHX chemoradiotherapy with reduced dose radiation in advanced head and neck cancer. *Clinical Cancer Research*.

[4] Vokes EE, Stenson K, Rosen FR (2003). Weekly carboplatin and paclitaxel followed by concomitant paclitaxel, fluorouracil, and hydroxyurea chemoradiotherapy: curative and organ-preserving therapy for advanced head and neck cancer. *Journal of Clinical Oncology*.

[5] Garden AS, Harris J, Vokes EE (2004). Preliminary results of Radiation Therapy Oncology Group 97-03: a randomized phase II trial of concurrent radiation and chemotherapy for advanced squamous cell carcinomas of the head and neck. *Journal of Clinical Oncology*.

[6] Vokes EE, Kies MS, Haraf DJ (2000). Concomitant chemoradiotherapy as primary therapy for locoregionally advanced head and neck cancer. *Journal of Clinical Oncology*.

[7] Bonner JA, Harari PM, Giralt J (2006). Radiotherapy plus cetuximab for squamous-cell carcinoma of the head and neck. *The New England Journal of Medicine*.

[8] Bonner JA, Harari PM, Giralt J (2010). Radiotherapy plus cetuximab for locoregionally advanced head and neck cancer: 5-year survival data from a phase 3 randomised trial, and relation between cetuximab-induced rash and survival. *The Lancet Oncology*.

[9] Lefebvre JL, Pointreau Y, Rolland F (2013). Induction chemotherapy followed by either chemoradiotherapy or bioradiotherapy for larynx preservation: The TREMPLIN randomized phase II study. *Journal of Clinical Oncology*.

[10] McGinn CJ, Shewach DS, Lawrence TS (1996). Radiosensitizing nucleosides. *Journal of the National Cancer Institute*.

[11] Richards GJ, Chambers RG (1969). Hydroxyurea: a radiosensitizer in the treatment of neoplasms of the head and neck. *The American Journal of Roentgenology, Radium Therapy, and Nuclear Medicine*.

[12] Vokes EE, Haraf DJ, Panje WR, Schilsky RL, Weichselbaum RR (1992). Hydroxyurea with concomitant radiotherapy for locally advanced head and neck cancer. *Seminars in Oncology*.

[13] Moran RG, Danenberg PV, Heidelberger C (1982). Therapeutic response of leukemic mice treated with fluorinated pyrimidines and inhibitors of deoxyuridylate synthesis. *Biochemical Pharmacology*.

[14] Eisenhauer EA, Therasse P, Bogaerts J (2009). New response evaluation criteria in solid tumours: revised RECIST guideline (version 1.1). *European Journal of Cancer*.

[15] Trotti A, Colevas AD, Setser A (2003). CTCAE v3.0: development of a comprehensive grading system for the adverse effects of cancer treatment. *Seminars in Radiation Oncology*.

[16] Haddad R, O'Neill A, Rabinowits G (2013). Induction chemotherapy followed by concurrent chemoradiotherapy (sequential chemoradiotherapy) versus concurrent chemoradiotherapy alone in locally advanced head and neck cancer (PARADIGM): a randomised phase 3 trial. *The Lancet Oncology*.

[17] Posner MR, Hershock DM, Blajman CR (2007). Cisplatin and fluorouracil alone or with docetaxel in head and neck cancer. *The New England Journal of Medicine*.

[18] Harwood AR, Beale FA, Cummings BJ (1981). T4M0N0 glottic cancer: an analysis of dose-time volume factors. *International Journal of Radiation Oncology∗Biology∗Physics*.

[19] Amornmarn R, Prempree T, Viravathana T, Donavanik V, Wizenberg MJ (1985). A therapeutic approach to early vocal cord carcinoma. *Acta Radiologica Oncology*.

[20] Schwaibold F, Scariato A, Nunno M (1988). The effect of fraction size on control of early glottic cancer. *International Journal of Radiation Oncology Biology Physics*.

[21] Kim RY, Marks ME, Salter MM (1992). Early-stage glottic cancer: importance of dose fractionation in radiation therapy. *Radiology*.

[22] Parson J, Million R, Cassisi N (1994). Time-dose-volume relationships in radiation therapy. *Management of Head and Neck Cancer: A Multidisciplinary Approach*.

